# Hearing impairment in the P23H-1 retinal degeneration rat model

**DOI:** 10.3389/fnins.2014.00297

**Published:** 2014-09-17

**Authors:** Jorge V. Sotoca, Juan C. Alvarado, Verónica Fuentes-Santamaría, Juan R. Martinez-Galan, Elena Caminos

**Affiliations:** ^1^Deparment of Medical Sciences, School of Medicine and Institute for Research in Neurological Disabilities (IDINE), University of Castilla-La ManchaAlbacete, Spain; ^2^Barn och UngdomsmedicinEskilstuna, Sweden

**Keywords:** ABR, cochlea, cochlear nucleus, hearing loss, immunocytochemistry, retinitis pigmentosa

## Abstract

The transgenic P23H line 1 (P23H-1) rat expresses a variant of rhodopsin with a mutation that leads to loss of visual function. This rat strain is an experimental model usually employed to study photoreceptor degeneration. Although the mutated protein should not interfere with other sensory functions, observing severe loss of auditory reflexes in response to natural sounds led us to study auditory brain response (ABR) recording. Animals were separated into different hearing levels following the response to natural stimuli (hand clapping and kissing sounds). Of all the analyzed animals, 25.9% presented auditory loss before 50 days of age (P50) and 45% were totally deaf by P200. ABR recordings showed that all the rats had a higher hearing threshold than the control Sprague-Dawley (SD) rats, which was also higher than any other rat strains. The integrity of the central and peripheral auditory pathway was analyzed by histology and immunocytochemistry. In the cochlear nucleus (CN), statistical differences were found between SD and P23H-1 rats in VGluT1 distribution, but none were found when labeling all the CN synapses with anti-Syntaxin. This finding suggests anatomical and/or molecular abnormalities in the auditory downstream pathway. The inner ear of the hypoacusic P23H-1 rats showed several anatomical defects, including loss and disruption of hair cells and spiral ganglion neurons. All these results can explain, at least in part, how hearing impairment can occur in a high percentage of P23H-1 rats. P23H-1 rats may be considered an experimental model with visual and auditory dysfunctions in future research.

## Introduction

The P23H mutant rhodopsin transgenic rat is an experimental model of retinal degeneration that exhibits gradual, fast photoreceptor loss with similar properties to human autosomal dominant retinitis pigmentosa (Berson et al., 1991; Lewin et al., 1998). Transgenic rats have a proline-23 to histidine (pro23His) *rhodopsin* mutation (LaVail et al., 2000; Aleman et al., 2001). Numerous studies have widely elucidated the morphology, function and molecular retina features of these animals to contribute to make progress in gene therapies, retina transplantation and alternative therapeutic approaches to slow photoreceptor degeneration (Machida et al., 2000; Aleman et al., 2001; Green et al., 2001; Zhang et al., 2003; Cuenca et al., 2004; Salzmann et al., 2006; García-Ayuso et al., 2010; Gorbatyuk et al., 2010; Kolomiets et al., 2010; Fernández-Sánchez et al., 2012; Jensen, 2012; Lu et al., 2013; Rahmani et al., 2013). There are three P23H mutant rat lines with different photoreceptor degeneration rates (http://www.ucsfeye.net/mlavailRDratmodels.shtml). The Line 1 animals have a higher level of transgene expression than the Line 3 animals and they have faster degeneration than the rats of Lines 2 and 3. Moreover, homozygous animals produce a faster degeneration rate than heterozygotes; indeed the retina of these animals has been studied in depth, but the rest of the central and peripheral nervous systems remains completely unexplored. In the present study, we evaluate the auditory capacity of the homozygous P23H line 1 (P23H-1) rats at different ages by using physiological and morphological techniques. Recent studies have found a direct relationship between rhodopsins and the auditory system (Shimano et al., 2013; Coleman et al., 2014). Rhodopsins virally targeted within auditory neurons of the dorsal cochlear nucleus had no detrimental effects on hearing and may be useful to modulate the activity of specific auditory neurons (Shimano et al., 2013).

The idea of testing the auditory system in P23H-1 rats emerged after observing a low response of these animals to natural stimuli (one single clap and kissing sounds). We decided to define the functional capabilities of specific components of the auditory system using auditory brain response (ABR) recordings. The components of the auditory pathway, analyzed by ABR, were those generally accepted in rodents: Cochlear Nerve (wave I); Cochlear Nuclei (wave II); Superior Olivary Complex (wave III); Lateral Lemniscus and/or Inferior Colliculus (wave IV); and Inferior Colliculus and/or Geniculated Body (wave V) (Simpson et al., 1985; Chen and Chen, 1991; Alvarado et al., 2012).

The ABRs results prompted us to evaluate whether physiological auditory alterations are also reflected in the morphological features of the central and peripheral auditory pathways. The cochlear nucleus (CN) of the auditory brainstem, the first relay station of the central auditory pathway, is a useful model system to achieve these goals (Benson et al., 1997). In particular, we examined the anteroventral subdivision of the CN (anteroventral CN, AVCN), where the cell bodies of the spherical and globular/bushy cells are localized. Spherical bushy cells receive direct input from the auditory nerve, called the endbulb of Held. One or two endbulbs reach the cell body of a spherical bushy cell which, in turn, transmits high-fidelity temporal information to structures in the superior olivary complex (Malmierca, 2003; Ryugo and Parks, 2003). Cochleae of P23H-1 rats were also analyzed. The organ of Corti, the modiolus and the stria vascularis are the three main cochlea structures. The organ of Corti contains inner and outer hair cells that amplify the acoustic signal and transform the mechanical signal into an electrical one. The modiolus contains bipolar spiral ganglion neurons which connect hair cells with the cochlear nucleus neurons in the brainstem through the auditory nerve. Finally, the stria vascularis contains epithelial and endothelial cells that maintain the ion composition of the endolymph (Raphael and Altschuler, 2003). These three cochlear regions are involved in hearing loss due to noise, age, ototoxic substances or inherited deafness (Li et al., 2013; Alvarado et al., 2014; El-Amraoui and Petit, 2014; Sun et al., 2014).

The main goal of the present study was to evaluate the hearing capabilities of mutant rhodopsin transgenic rats P23H-1. Accordingly, we hypothesize that severe loss of auditory reflexes in response to natural sounds in P23H-1 rats might be the result of structural and functional alterations along peripheral and central pathways. To investigate this possibility, the integrity of the inner ear and the cochlear nucleus, the first relay station of the auditory pathway, was investigated in P23H-1 rats in comparison to Sprague-Dawley animals. Our results provide evidence that the P23H-1 homozygous transgenic rat represents an excellent animal model to study visual and auditory dysfunctions. While P23H rats are widely used to study photoreceptor degeneration, in the auditory system, they could be used to understand the specific function of neurons in hearing because it is known that rhodopsin can mediate neuronal activity in auditory neurons (Shimano et al., 2013).

## Materials and methods

### Animals

Transgenic P23H-1 homozygous albino rats for breeding were kindly provided by Dr. Matthew LaVail (UCSF School of Medicine, Beckman Vision Center, San Francisco, CA, USA), and were bred in a colony at the University of Castilla-La Mancha. As wild-type controls, Sprague-Dawley (SD, Charles River Laboratories, Barcelona, Spain) were used. All the animals were housed and handled according to the authorization and supervision of the Animal House Facility of the University of Castilla-La Mancha, after receiving approval from the Ethics Committee for Experimental Animal Welfare of the University of Castilla-La Mancha (CEEA). These studies were conducted in accordance with the guidelines of the European Council (Directive 2010/63/UE) and current Spanish regulations (R.D. 1201/2005 and P.L. 121/000123/2007) the use and care of animals in research.

### Auditory testing

Auditory stimuli were presented in a normal laboratory environment in the animal house. Rats were stimulated with two natural sounds: single clap (SC) and kissing sounds (KS).

Singly housed animals were tested while still in their cages with the lid removed and were exposed to one SC. Then, the rat were exposed to two short KS. Approximately, 2 min were spent to evaluate each animal once per week. Animals were scored in the ears depending on the observed response.

In order to generate a standard test battery, these sounds were recorded with a sound level meter, model VOLTCRAFT SL-200 (Ventus, Madrid, Spain). Stimuli were always run by the same person and in the same room with no other sound disturbance. SD and P23H rats lived and were stimulated under the same conditions. The intensity of a SC fell within the 85–95 dB range, with a frequency range between 1 and 2.5 kHz, while that of the KS ranged between 60 and 65 dB, with a frequency range between 1.1 and 7.2 kHz. The response to these sounds was evaluated in 54 P23H-1 rats (from P20 to P200, or older) and in two SD rats (P80 and P112). All the P23H-1 rats came from one breeding pair of homozygous P23H-1 rats and from crossings of first generation members. The auditory response consisted in observing the whole body startle response and Preyer's reflex (Jero et al., 2001). Animals responded to the KS by displaying a rapid movement of the whole body and then remained motionless with their head up for a split second (“alert movement”) before they continued with their exploratory movements. The response to the SC was a rapid movement of the whole body, but the animal did not remain still, but continued making exploratory movements, with their head down and smelling the box. Each animal was ranked based on its response to these gross auditory assessments at three auditory capacity levels. Level I: rats responded to both the SC and KS with fast reflexes (considered to be normal audition); Level II: rats responded only to the KS by displaying slow alert movements; Level III: animals did not respond to either stimuli.

### Auditory brainstem response (ABR)

To apply more objective measures to define the functional capabilities of the P23H rat auditory system, ABRs measurements were analyzed in 17 rats after the first assessment of auditory response to the above-described natural sounds. Fifteen P23H-1 transgenic rats were selected which presented different levels of audition subsequently to the auditory test results (Levels I–III) and at several postnatal ages (P20; P52; P56; P80; P100; P133; P180). Two control SD rats (aged P80 and P112), classified as Level I, were used during the gross hearing assessment.

The ABRs were performed as previously described (Alvarado et al., 2012; Lamas et al., 2013). Briefly, animals were located in a sound-attenuating, electrically shielded booth (EYMASA/INCOTRON S.L., Barcelona, Spain) and were placed inside a sound-attenuating room. Anesthesia was induced (4%) and maintained (1.5–2%) with isoflurane (1 L/min O_2_ flow rate). Subdermal electrodes (Rochester Electro-Medical, Tampa, FL, USA) were placed at the vertex (non-inverting) and under the right (inverting) and left (ground) ears. Acoustic stimulation and recordings were performed with a Tucker-Davis (TDT) BioSig System III (Tucker-Davis Technologies, Alachua, FL, USA). Stimuli consisted in tone bursts (5 ms rise/fall time, no plateau, cos2 envelope, at 20/s) at seven different frequencies (0.5, 1, 2, 4, 8, 16, and 32 kHz), which were generated digitally with the SigGenRP software (Tucker-Davis Technologies) and the RX6 Piranha Multifunction Processor hardware (Tucker-Davis Technologies). Stimuli were delivered into the external auditory meatus in the right ear using an EDC1 electrostatic speaker driver (Tucker-Davis Technologies) through an EC-1 electrostatic speaker (Tucker-Davis Technologies). Throughout the procedure, rat temperature was monitored by a rectal probe and was maintained at 37.5 ± 1°C with a non-electrical heating pad. Prior to the experiments, stimuli were calibrated by the SigCal software (Tucker-Davis Technologies) and an ER-10B+ low-noise microphone system (Etymotic Research Inc., Elk, Groove, IL, USA). The evoked potentials were filtered (0.3–3.0 kHz), averaged (500 waveforms) and stored for offline analyses.

In order to determine the auditory threshold level, evoked responses were recorded in 5 dB steps, which descended from a maximum stimulus intensity of 80 dB SPL. The auditory threshold was defined as the stimulus intensity that evoked waveforms with a peak-to-peak voltage higher than 2 standard deviations of background activity (measured before stimulus onset).

### Immunofluorescence procedure in the cochlear nucleus

The integrity of the auditory synapses was analyzed by immunocytochemistry in the first relay station of the central auditory pathways, the AVCN, where output and input from ascending and descending pathways are present. At the end of the recording sessions, six animals (2 SD and 4 P23H rats) were anesthetized with ketamine (100 mg/kg, Parke-Davis, Alcobendas, Spain) and 2% xylazine (10 mg/kg, Dibapa, Barcelona, Spain) and were transcardially perfused with 0.9% saline and 2% paraformaldehyde in 0.1 M phosphate buffer (PB), pH 7.3. Brains were removed, postfixed for 4 h in the same fixative, washed in 0.1 M PB, transferred into PB containing 30% sucrose, and embedded in Tissue Tek (Leica, Wetzlar, Germany). Serial sections were obtained at 16 μm with a cryostat (Leica), mounted onto Super Frost slides (Kindler, Freiburg, Germany) and used for immunocytochemistry.

Single immunofluorescence procedures were performed as previously described (Caminos et al., 2007) to test the presence of VGluT1 and Syntaxin in the AVCN synapses of the control rats (*SD, n* = 2, P80 and P112), P23H rats with ABR waves (Level I, *n* = 2, P52 and P80), and P23H rats with profound deafness (Level III, *n* = 2, P52 and P100). Cryosections were washed in phosphate-buffered saline, pH 7.3 (PBS), containing 0.25% Triton X-100 (PBST), and were pre-incubated for 1 h at room temperature (RT) with blocking solution containing PBST and 1% BSA (Fraction V, Sigma-Aldrich, Steinheim, Germany). Sections were then incubated with primary antibodies to recognize type I vesicular glutamate transporter (mouse anti-VGluT1 monoclonal antibody, 1:500, clone N28/9, NeuroMab, Davis, CA, USA) and Syntaxin (mouse anti-Syntaxin monoclonal antibody, 1:1000, clone HPC-1, Sigma, Germany). Antibodies were diluted in PBST-BSA overnight at RT in a humid chamber and were then washed with PBST. Immunoreactivity was visualized using a secondary anti-mouse antibody coupled to Cy5 (1:200; Jackson ImmunoResearch, West Grave, PA, USA) for 1 h at RT. Finally, brain sections were washed in PBST, air-dried in the dark and mounted with Duolink *In Situ* Mounting Medium with DAPY (Olink Bioescience, Uppsala, Sweden). Immunofluorescence sections were examined under a Zeiss LSM 710 laser scanning confocal microscope (Zeiss, Germany). Images were analyzed using the ZEN 2009 Light Edition software (Zeiss) and the Image-J software (Rasband, W.S. National Institutes of Health, Bethesda, MD, USA, http://rsb.info.nih.gov/ij/). The immunolabeling controls included (1) incubation with a primary antibody followed by a conjugated secondary antibody which did not recognize the host species in which the corresponding primary antibody was obtained; (2) omission of the primary antibody. Neither control showed specific labeling.

For the statistical analysis, the digital Z-axis image stacks of the VCN were acquired from each brain section (14–16 sections/animal) under the confocal microscope using a Plan-Apochromat 20x/0.8 M27 objective. Images size was 424.68 × 424.68 μm and resolution was 1024 × 1024 (8-bit). All the confocal settings were the same for the analyses across all the analyzed images. From each stack, the image with the greatest red-fluorescence intensity was automatically selected by the confocal software. Each confocal microscope image was separated into two images: one with red immunostaining and another with blue nuclei stained with DAPI. These selected images were used for the quantitative assessment of immunoreactive profiles for VGluT1 or Syntaxin with the Image-J software. In a first step, the scale bar was calibrated at a known distance to select the size of the immunofluorescence particles to be counted. Then each image was converted into a grayscale 8-bit and was then transformed into a “binary image.” The red-fluorescence particles with a minimum size of 5 and 20 μm were counted. In addition, the nuclei or the blue-fluorescence particles with a minimum size of 5 μm were counted to determine the total number of cells. The statistical analysis was performed with the ratio fluorescent particles/number of nuclei per section. Data are presented as the mean ± Standard Deviation. The statistical analysis was performed by a Kruskal-Wallis test with a subsequent pair-wise comparison made using the Mann-Whitney *U*-test.

### Cochlear histology and immunocytochemistry

Animals were anesthetized and perfused as indicated above. Cochleae were quickly removed, postfixed in 4% paraformaldehyde for 2 h and decalcified in 10% ethylenediamine tetraacetic acid (EDTA; pH 6.5) solution for 10 days. The left side was used for cochlear sensory epithelia surface preparations and the right side for myosin VIIa immunocytochemistry and Nissl staining.

#### Whole mount immunocytochemistry

Each turn of the organ of Corti was detached from the modiolus and the sensory epithelium incubated overnight with rabbit anti-myosin VIIa primary antibody (1:100; Proteus Biosciences Inc., Ramona, CA, USA). The following day, the tissue was incubated for 2 h at RT in an anti-rabbit Alexa Fluor 594 secondary antibody and Alexa Fluor 488-conjugated Phalloidin (Invitrogen-Molecular Probers, Carlsbad, CA, USA) and was mounted with DAPI nuclear staining.

#### Myosin VIIa immunocytochemistry

Cochleae were embedded in 10% gelatin, and were oriented in such a way that the modiolus was parallel to the base and frozen at −70°C by immersion in a solution of 2-propanol. Cochleae were sectioned at 20 μm on a cryostat and mounted onto Super Frost slides. To detect myosin, sections were rinsed in PBS containing 0.3% Triton X-100 (Tx) and blocked for 1 h in PBS-Tx (0.2%) containing 10% normal goat serum (NHS). The first series of sections was incubated overnight at 4°C with myosin VIIa antibody in a solution containing PBS-Tx (0.2%), pH 7.4. Then, sections were washed 4 × 15 min in PBS-Tx (0.2%), and incubated for 2 h in biotinylated anti-rabbit secondary antibody (1:200; Vector Laboratories, Burlingame, CA) and for 1 in the avidin-biotin-peroxidase complex solution (ABC, Vector lab). Finally, sections were mounted onto gelatin-coated slides and coverslipped using Cytoseal (Richard-Allan Scientific, Kalamazoo, MI, USA). The second series of sections was stained with cresyl violet.

## Results

### Response to gross auditory assessment

The first aim of this study was to determine whether the response of P23H rats to auditory natural stimuli differed from that of the control SD rats. While all the SD rats responded to the SC and KS, not all the P23H rats responded to these stimuli. We classified animals into three levels based on the observed response to these gross auditory assessment: Level I, a normal hearing level, where rats responded to both the SC and KS stimuli; Level II, an intermediate hearing level, where rats responded only to the KS stimulus, and never to the SC one (lower frequency and higher intensity noise than the KS). Finally, Level III included animals with profound deafness, which meant that they did not respond to any sound.

We analyzed the response to natural stimuli in all the offspring of five couples of P23H-1 rats: (1) one breeding pair with a deaf male and a deaf female at age P100 (both Level III); (2 and 3) two breeding pairs of a normal male and a normal female at age P100 (Level I); (4) one male and female pair with Level II at P100; (5) one pair with a Level I male and a level II female at P100. All the pairs, except (3), had offspring with auditory responses at Levels I–III, regardless of their parents' hearing status. All the litters of pairs (1), (2), and (5) were totally viable and all the offspring survived. Nevertheless, only one litter of pair (4) survived, while no offspring of pair (3) survived. Thus, we cannot state that the survival of these P23H-1 rats depended on parents' auditory capacity. In fact, all the pairs, except (3), had offspring with auditory responses at Levels I–III, regardless of their parents' hearing status.

The auditory test was applied to all the descendants aged from P20 and it was repeated several times during their life until the animal was sacrificed (at P20–P60, P90–P110, P160–P200 and older). We found that most rats responded to both the KS and SC (Level I), some responded to only one stimuli and displayed slow alert movements (Level II), while others did not respond to any sound (Level III), even at P20 (Table 1). As these responses were variable during animals' life, we can state that auditory capacity diminished with age. Table 1 includes the percentage of rats at the different auditory levels based on their response to natural stimuli. These data reflect progressive auditory capacity loss during the life of P23H-1 rats. Progression to deafness was evident from P100 onward, because 55% of normal rats (Level I) at P20 belonged to Level II at P100, and 45% of the oldest animals were profoundly deaf (Level III) at the age of P200.

**Table 1:** **Percentage of the P23H-1 rats classified into the three hearing levels based on their response to natural sounds at different ages**.

**Age**	**P20–P60**	**P90–P110**	**≥P160**
Level I	74.1	44.5	27.5
Level II	14.8	40.7	27.5
Level III	11.1	14.8	45
	*n* = 54	*n* = 54	*n* = 40

*Level I, considered “normal hearing” (response to the KS and SC sounds); Level II, a response only to the KS; Level III, considered deaf (no response to either stimuli); n, number of animals analyzed*.

Of all the young (P20–P60) rats, 25.9% presented moderate hearing loss, with the remaining 11.5% corresponding to the profound deafness group (Table 1). These numbers increased with age, mainly from P100, with 45% of the older adult rats not responding to either sound that they were tested for. The results of this hearing test were corroborated when some animals were subjected to ABR recordings.

### Auditory brainstem response (ABR)

**Figure 1:**
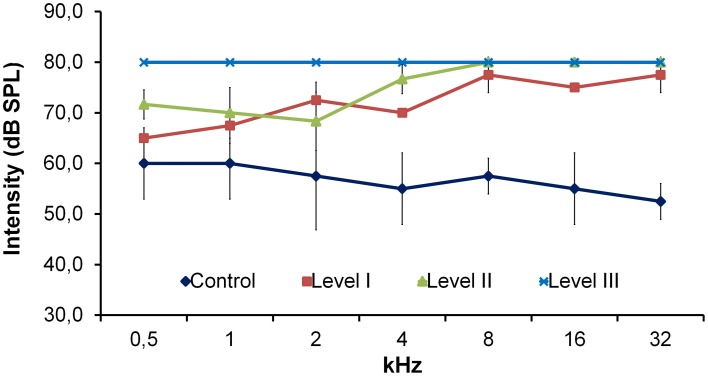
**Line graph showing the relationship between auditory thresholds and the frequency of the stimuli evaluated in the control SD rats (*n* = 2) and P23H rats**. P23H rats were distributed according to their responses to natural stimuli into: Level I (*n* = 2), Level II (*n* = 3), and Level III (*n* = 10). The auditory threshold in P23H-1 rats was always higher than in SD rats. Data are expressed as mean ± standard deviation.

ABRs were recorded in 15 selected P23H-1 rats of different ages (P20; P52; P56; P80; P100; P133; P180) which corresponded to the three audition levels based on their responses to natural stimuli (see above), and in two control SD rats (P80 and P112). The P23H recordings were always compared with the ABR recordings of the control SD rats, which had the lowest auditory thresholds considered with normal audition (Burkard et al., 1990; Newton et al., 1992), as seen in Figure 1. Level I P23H-1 rats achieved the lowest auditory thresholds, which were always higher than those of the control rats. Level II P23H rats could hear a low frequency, but were totally deaf at a high frequency. P23H rats with audition Level III presented profound deafness at any frequency (Figure 1).

**Figure 2:**
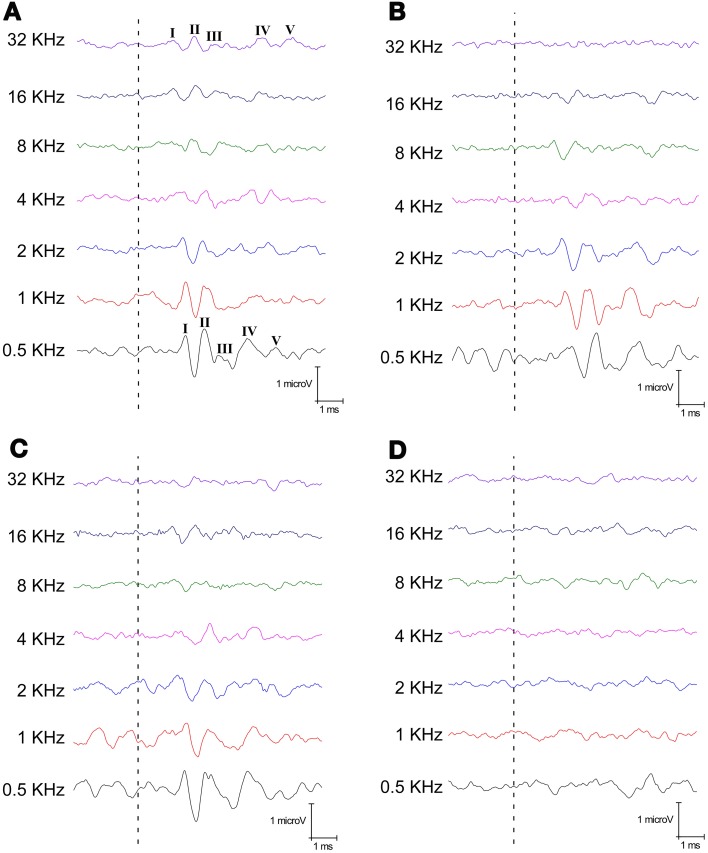
**Examples of ABR recordings from the control SD and P23H-1 rats at 80 dB SPL for all the frequencies tested. (A)** A control SD rat at P112, when the five waves are evident at any frequency. **(B)** A P23H rat at P52 that responded to both natural stimuli (kissing sounds and one single clap; Level I). **(C)** A P23H rat at P80 that responded only to the kissing sound as a natural stimulus (Level II). Note that the waves are evident at low and intermediate frequencies in both examples **(B,C)**, but these waves lost their morphology at higher frequencies (4–8 kHz). **(D)** A P23H rat at P180, when the ABR totally lost the waveform, even at the lowest frequencies. Dashed lines indicate stimulus onset.

Consistently with previous studies in rodents, the ABR of the SD rats displayed the five typical evoked waves at the different frequencies evaluated (Figure 2A) with wave II being the largest of all the waves comprising the ABR recordings (Overbeck and Church, 1992; Church et al., 2010, 2012; Alvarado et al., 2012, 2014). Although morphology was similar in Level I P23H rats to that observed in SD rats at the low and intermediate frequencies, the amplitude of all waves diminished at higher frequencies (Figure 2B). At level II, the amplitude of the waves across the frequencies also diminished for P23H, but became more evident at the higher one (Figure 2C). Finally for Level III P23H, which did not respond to either gross auditory assessment, the evoked waves at all frequencies evaluated were totally absent (Figure 2D). These results corroborate those obtained by applying the gross auditory assessment and show limitations in the normal auditory function of P23H rats.

### VGluT1 and syntaxin immunofluorescence in the VCN

**Figure 3:**
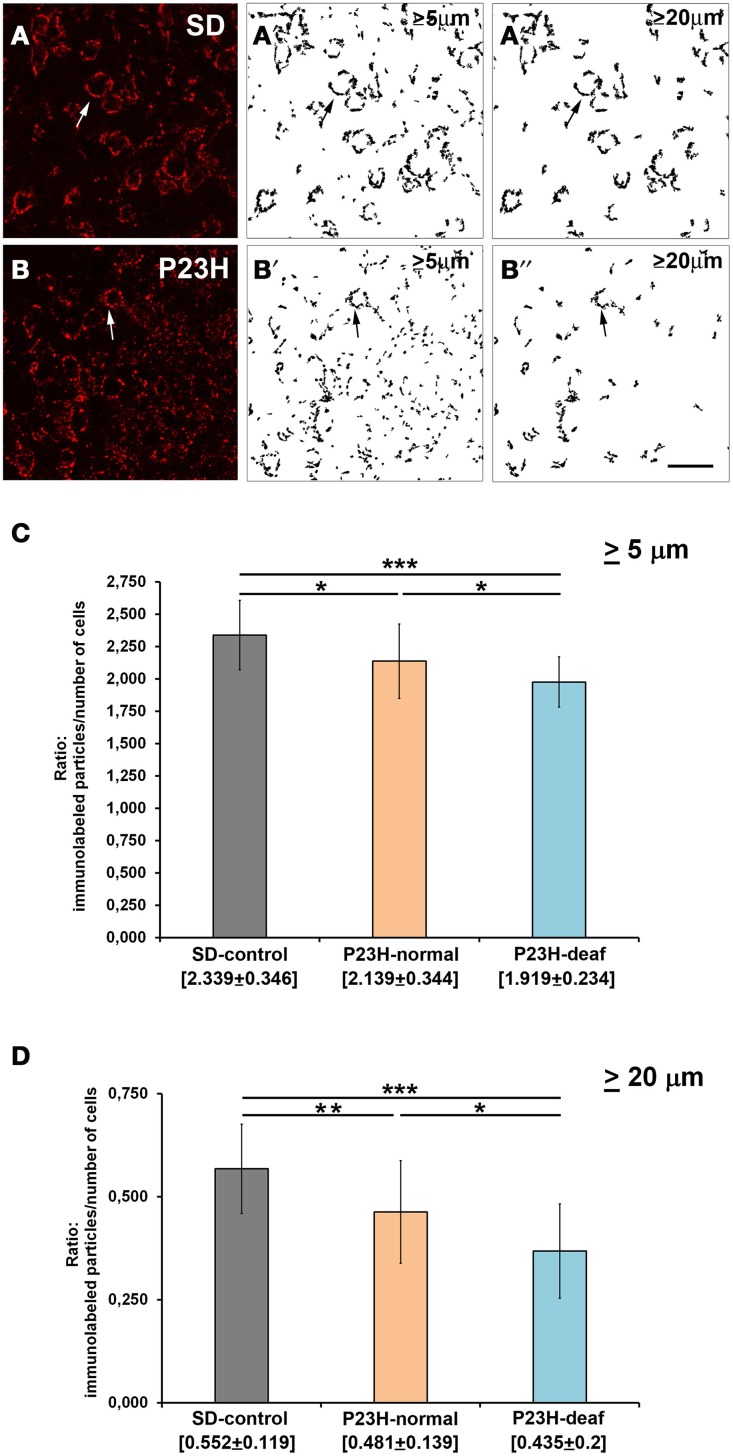
**VGluT1 immunolabeling in the coronal sections of the ventral cochlear nucleus of the control SD and P23H-1 rats**. Stained endbulbs of Held were red around cell bodies (arrows) in SD rats **(A)** and in deaf P23H-1 rats **(B)**. (**A′**,**A″**,**B′**,**B″**) Rendering the labeling for the binary image analysis obtained with the ImageJ software, and counting the labeled subcellular particles qualified by size [from 5 μm to infinity in **(A′,B′)**; and from 20 μm to infinity in **(A″,B″)**]. **(C,D)** Balance of the VGluT1 positive particles in the ventral cochlear nucleus of P23H rats (deaf and with normal hearing) as compared with the control group (SD). Statistical differences were found when the counted particles were larger than 5 μm **(C)**, and significant differences also appeared when the immunolabeled particles were larger than 20 μm **(D)**. Data are expressed as means ± standard deviations, and the levels of significance are indicated as ^***^p ≤ 0.001; ^**^p ≤ 0.05; ^*^p < 0.1 or 0.2. Scale bar: 50 μm.

VGluT1 immunostaining under confocal microscopy revealed dense punctate immunostaining of the glutamatergic presynaptic terminals in the AVCN of both SD and P23H-1 transgenic rats (Figures 3A,B). A visual assessment of VGluT1 labeling showed an apparent difference in the distribution of VGluT1 between SD and P23H rats. The endbulbs of Held in SD rats were completely labeled and surrounded the bushy cell soma, while punctate VGluT1 immunolabeling was found in the endbulbs of the deaf P23H-1 rats. Accordingly, a statistical analysis was performed on the binary images of the VCN of the control SD rats (Figures 3A′,A″), the P23H rats with normal hearing and the deaf P23H rats (Figures 3B′,B″). Significant differences were found in the number of immunofluorescence particles larger than 5 μm between the SD and deaf P23H rats (*p* < 0.001) (Figure 3C). Significant differences were also observed between the SD and P23H rats with normal hearing (*p* < 0.1), and between the deaf and normal P23H rats (*p* < 0.2), but levels of significance were lower (Figure 3C). When the fluorescent particle size was bigger than 20 μm (Figure 3D), significant differences were also detected between not only the SD and deaf P23H rats (*p* < 0.001), but also between the SD and P23H-1 rats with normal hearing (*p* < 0.05) and the deaf P23H and normal P23H rats (*p* < 0.1). The number of cell nuclei (neurons and glial cells) labeled with DAPI in the AVCN of the control SD, the P23H rats with normal hearing and the profound deaf P23H rats was similar for them all, and no statistically significant differences were seen. It is common not to find remarkable morphological changes in the CN if we consider that when the auditory system approaches maturity after hearing onset, and even in the adult brain, little or no neuronal loss occurs following cochlear injury (Mostafapour et al., 2000; Hildebrandt et al., 2011).

**Figure 4:**
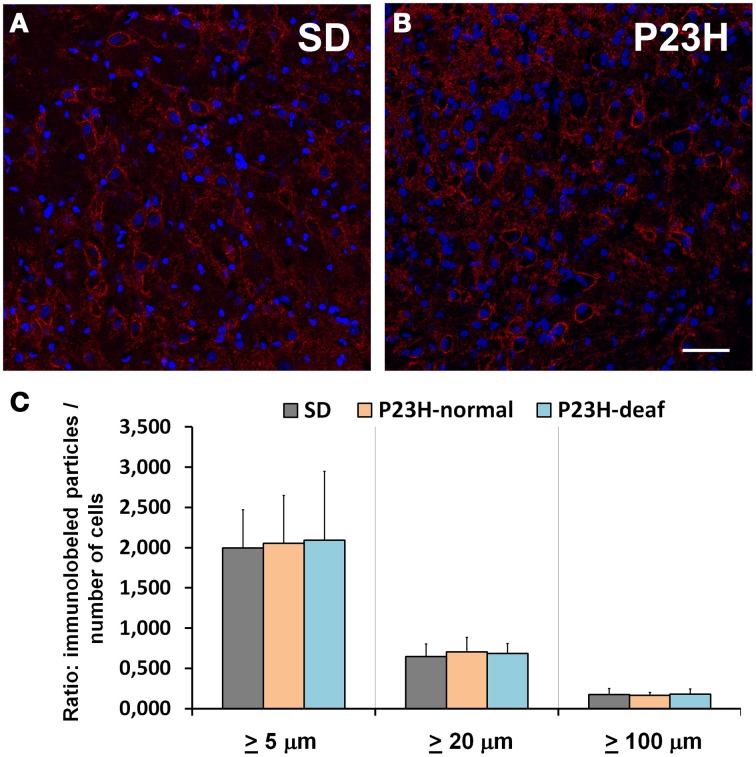
**Syntaxin immunolabeling in the coronal sections of the ventral cochlear nucleus of the control SD (A) and P23H (B) rats**. Histogram representing the balance of the fluorescence particles following the method described above **(C)**. No statistical differences were found when analyzing the three immunolabeled particles sizes: larger than 5, 20, or 100 μm. Scale bar: 50 μm.

Syntaxin immunolabeling distribution was also analyzed in the AVCN of the control SD and P23H-1 rats with normal hearing (Level I) and in the deaf (Level III) ones (Figures 4A,B). A statistical analysis was done with the binary images as in the VGluT1 analysis. No statistical differences were found in the number of syntaxin immunoreactive particles in any comparison made (Figure 4C).

### Cochlear abnormalities in P23H-1 rats

**Figure 5:**
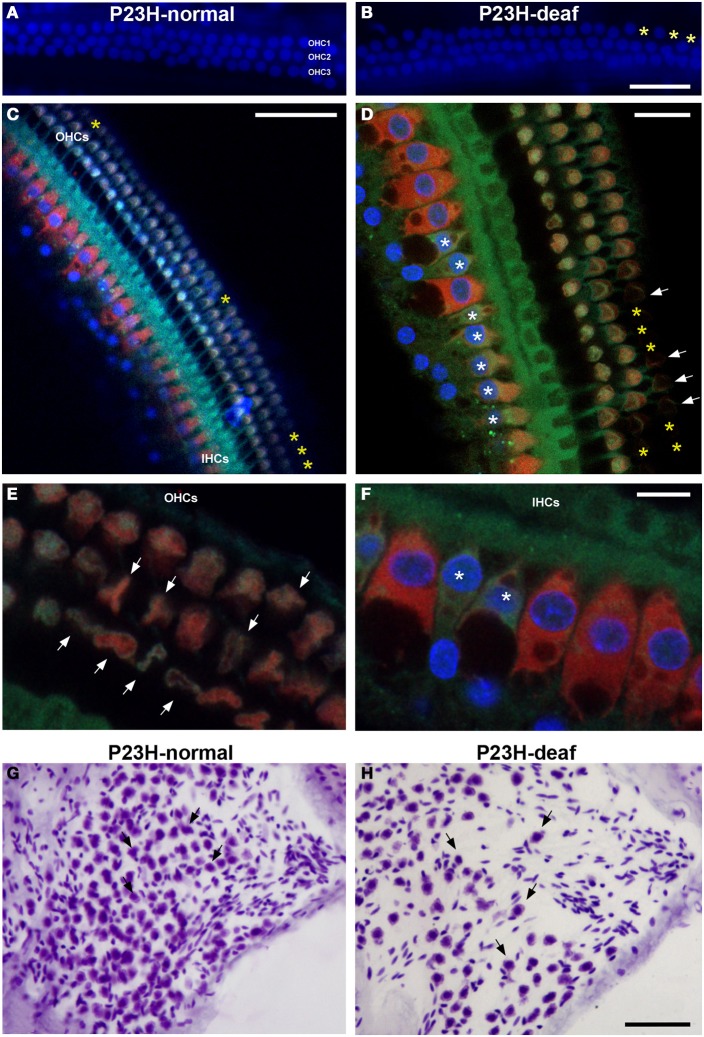
**Cochlear histology in P23H-1 rats**. Confocal images of surface preparations from the middle cochlear turn of P23H-1 rats with normal ABR recordings (**A**, P23H-normal) and hypoacusic P23H-1 rats **(B–D)**. Loss of outer hair cells in the hypoacusic P23H-1 rats (yellow asterisks, **B–D**) as compared to the control rats **(A)** is observed. Note the abnormal morphology of the outer (arrows in **D**) and inner (asterisks in **D**) hair cells in the hypoacusic animal. High magnification images of these alterations are shown in **(E,F)**. Disruption of outer **(E)** and inner **(F)** hair cells are indicated by arrows and asterisks, respectively. Spiral ganglion cells in the hypoacusic P23H-1 rats were slightly larger with decreased in density **(H)** when compared to the control animals **(G)**. Filamentous actin was stained with Phalloidin (green), while myosin staining is shown in red. DAPI was used as nuclear staining (blue). IHCs, inner hair cells; OHCs, outer hear cells. Scale bars = 20 μm in **(B,D)**; 50 μm in **(C,H)**; 10 μm in **(F)**.

**Figure 6:**
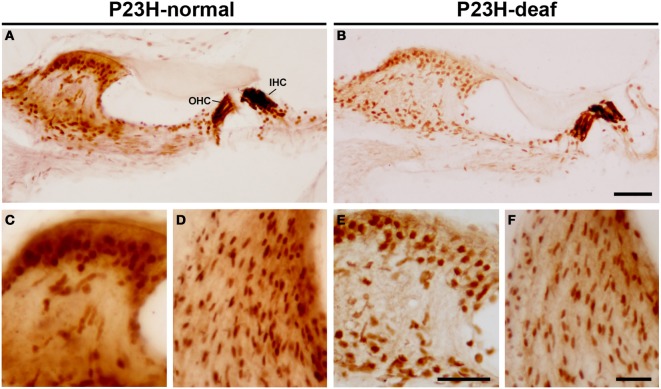
**Myosin immunostaining in the inner ear of P23H-1 rats**. There was a reduced myosin immunostaining in the Organ of Corti **(B)**, spiral limbus **(E)** and stria vascularis **(F)** of the hypoacusic P23H-1 rats in comparison to P23H-1 rats with normal ABR recordings **(A,C,D)**. IHC, inner hair cells; OHC, outer hear cells. Scale bars = 50 μm in **(A,B)**; 25 μm in **(C–F)**.

To determine whether the functional deficit observed in the ABR recordings of P23H-1 rats was accompanied by a cochlear pathology, we first evaluated the integrity of hair cells in these animals (Figures 5A–F). The results demonstrate loss (yellow asterisks in Figures 5B–D) and disruption (arrows in Figure 5E) of the outer hair cells in the hypoacusic P23H-1 rats when compared to the control animals (P23H-1 rats with normal ABR recordings) (Figure 5A). Although there was no apparent loss of inner hair cells in the hypoacusic animals, these cells were disorganized and had shortened cell bodies (asterisks in Figures 5D,F). The cytoarchitecture of the spiral ganglion cells was also investigated and revealed that these cells were larger and smaller in number (arrows in Figures 5G,H). Along with these changes, myosin VIIa expression was investigated in the cochlea of P23H-1 rats to determine possible alterations in the function of this protein. Myosin VIIa immunocytochemistry revealed that myosin filamentous levels were low in the inner and outer hair cells of hypoacusic rats (Figures 6A,B) and also in other cochlear structures including the spiral limbus (Figures 6C,E) and the stria vascularis (Figures 6D,F), when compared to the control animals (Figures 6A,C,D). Additional anatomical defects in the inner ear of P23H-1 rats included a physical attachment of the tectorial membrane to the sensory epithelium (Figure 6B), as has been observed in animal models of sensorineural deafness (Camarero et al., 2001).

## Discussion

All the assays in this study confirm that P23H-1 rats have a significant hearing deficit. It is noteworthy that the auditory threshold in all P23H-1 rats was higher than that in the control SD rats and in any other rat strain generally used in animal research. Our observations are supported by other studies conducted with ATP8A2 transgenic mice. Interestingly, the rhodopsin content in these mice is decreased, the ABR threshold is higher and the spiral ganglion cells appear to be degenerated (Coleman et al., 2014).

### Physiological findings

The transgenic rhodopsin P23H mutant rat is an experimental retinal degeneration model that is widely used to study retinitis pigmentosa. These rats are in the albino Sprague-Dawley (SD) background, thus SD rats are employed for wild-type controls. Homozygous P23H-1 rats are mainly used to provide early information about retinal degeneration. Here we evaluate the auditory capacity of this strain. Our preliminary observations detected a considerable number of P23H-1 rats with no impairment to respond to gross auditory assessment such as the KS and SC. In some cases, such deficiencies appeared at an early age and progressed during the rat's lifetime to reach profound deafness. The first auditory test was done at P20 when the central auditory system is considered mature. In other cases, auditory alterations began later, at around P100, and some animals were totally deaf at P200. In order to organize the hearing test results, we divided offspring into three audition levels based on their responses to natural stimuli: Level I corresponds to P23H-1 rats, which responded to the KS and SC stimuli; Level II corresponds to those animals which responded only to the KS; Level III corresponds to the animals that responded to neither the KS nor the SC. With these preliminary results, we were able to predict progressive hearing loss in some P23H-1 rats.

The auditory test analysis was corroborated by the ABR recordings. The ABR is a commonly used technique to study auditory function under either normal or pathological conditions. In all the P23H rats evaluated, the auditory thresholds were always higher than those observed in SD rats, even when P23H rats responded to both KS and SC sounds (Level I). The amplitude for all the waves also diminished, which became more evident as the animal Level became higher; thus in the P23H-1 rats at Level III, the obtained ABR recordings were totally flat. Since it is generally accepted that waves I–V correspond to activity from the cochlea to the IC (Overbeck and Church, 1992; Church et al., 2010, 2012; Alvarado et al., 2012), the fact that in P23H the amplitude of all waves diminished suggests diverse anatomical abnormalities along the auditor pathway in this rat model. The alterations noted in the Organ of Corti, the spiral ganglion and the CN could explain the reduction noted in the amplitude of waves I and II in P23H-1 rats. Based on the high auditory thresholds and the differences in the ABR waves, we suggest that P23H-1 rats have altered auditory functional capabilities if compared to other rat strains, like SD, Long-Evans, Wistar or Fisher rats (Burkard et al., 1990; Newton et al., 1992; Overbeck and Church, 1992; Polak et al., 2004). Similarly, the retinal ganglion cells of the degenerated retina of P23H-1 rats have higher thresholds than normal retinas (Jensen and Rizzo, 2011; Jensen, 2012).

Progression of deafness with age in the homozygous P23H-1 rats resembled photoreceptors degeneration progression (Machida et al., 2000; Cuenca et al., 2004); http://www.ucsfeye.net/mlavailRDratmodels.shtml; our own studies). Photoreceptor cell loss begins at around P20. At P90, there are 2–3 layers of photoreceptors, and between P180 and P200, all the photoreceptors have almost disappeared and the rat is blind. In our study, hearing loss was also progressive in most rats, with more rapid loss from P100, and they became completely deaf at P200. In general, all the mammals present age-associated hearing loss. Nonetheless, a P180-aged experimental rat is considered young to have such auditory disabilities under normal conditions and if compared with other rat strains and other murine models (Borg, 1982; Schweitzer, 1987; Burkard et al., 1990; Cooper et al., 1990; Chen and Chen, 1991; Newton et al., 1992; Overbeck and Church, 1992; Church and Kaltenbach, 1993; Polak et al., 2004; Alvarado et al., 2012). Current optogenetic studies support the possibility that hearing impairment in P23H-1 is a direct effect of the rhodopsin mutation. Rhodopsin-mediated neuronal activity in the dorsal cochlear nucleus neurons of mice transfected with opsins adeno-associated viral vectors has been demonstrated (Shimano et al., 2013). In these mice, optical stimulation *in vivo* resulted in neuronal activity in the dorsal cochlear nucleus. Thus, the incorporation of rhodopsin into auditory neurons can be used as a tracer of auditory pathways to understand the specific function of neurons in hearing (Shimano et al., 2013). It is important to note that not all the P23H-1 rats in our study developed deafness, even if their hearing thresholds were higher than those in SD rats. In contrast, some were deaf at P20. Given the technical limitation of the ABR equipment, it is not possible to know if these last animals were born deaf.

### Integrity of the cochlea and the cochlear nucleus synapsis

Initially, we considered that auditory alterations in P23H-1 rats could be of both a peripheral and central origin. We evaluated both possibilities by running an immunocytochemistry analysis of two proteins, which are widely distributed in the synaptic endings in the CN. Syntaxin is a protein located in all the synapses from the up- to downstream pathways reaching the CN, while VGluT1 is restricted to excitatory terminals on CN neurons (Garcia-Pino et al., 2010; Fyk-Kolodziej et al., 2011), mainly in the endbulbs of Held in the AVCN. In this study, we found no significant differences in the immunodistribution of syntaxin between the control SD and P23H-1 rats. However, the statistical analysis of anti-VGluT1 immunolabeling showed significant differences between SD rats and P23H rats, which were deaf or not deaf. To support these observations, VGluT1 expression was not detected in large auditory nerve terminals of VCN neurons 3 days after cochlear injury in rat (Fyk-Kolodziej et al., 2011). Our results suggest that the anatomical and/or molecular organization of the endbuld of Held in P23H-1 rats could differ from other rats with no hearing loss and, consequently, the anatomical origin of hearing loss can lie in the peripheral auditory pathway. Our results demonstrate that the hypoacusic P23H-1 rat displays multiple alterations in the inner ear, including loss and disruption of hair cells and spiral ganglion neurons, if compared with rats with normal ABR recordings. These morphological anomalies, along with the functional alterations described herein, demonstrate an increase in their auditory thresholds and a reduction in the amplitude of all the waves, which support the notion that these animals suffer from auditory dysfunction which results in affected neurotransmission in the cochlear nucleus.

The P23H-1 rat experimental model is very important to study retinal degeneration and can imply progressive loss of hearing function, as we show herein. We cannot assume a compensatory mechanism to visual impairment which promotes sense of hearing, unlike what happens in mice and cats with visual deprivation (Rauschecker et al., 1992; Rauschecker and Korte, 1993). This work opens up other fields of study into pathologies in which both the visual and auditory systems are implicated.

### Survival considerations

P23H mutant rats showed no breeding difficulties in the animal house, following the guidelines provided by Dr. M. LaVail. However, there were some cases of cannibalism of pups by the breeding pair. Deafness can be a barrier for parenting, as has been demonstrated in some strains of mutant mouse models. For example, mice with a homozygous mutation in cadherin 23 (C57BL/6J-Cdh23v-2J/J) are deaf from birth, they have no maternal instinct and their pups usually die (information from The Jackson Laboratory, and our own observations). For this reason, we wondered whether there was a relation between survival of pups and parents' auditory capacity. In this study, we found that parents with normal hearing can have offspring with impaired hearing, and that totally deaf parents can bear offspring with normal hearing. Therefore, breeding pairs with normal hearing is no guarantee that all offspring will have normal hearing. Although the success of offspring survival does not depend on parents being deaf, it is commonplace to find old breeding pairs with profound deafness when cases of cannibalism occurred.

## Conclusions

In the present report, we demonstrate that the transgenic P23H-1 rats employed to study retinal degeneration also underwent hearing deficiencies, which may appear at early age and can progress during lifetime to profound deafness. P23H-1 rats have a higher hearing threshold than the control SD rats and a higher one than other rat strains commonly used in research. Rhodopsin deficiency can be the molecular origin of auditory impairment in P23H-1 rats. Morphologically, the Organ of Corti and the spiral ganglion are the main affected structures. Consequently, these anatomical defects would result in altered excitatory inputs to the CN, which is reflected in the ABR recordings as abnormal auditory thresholds and reduced wave amplitudes. The results presented herein provide sufficient data to consider that a detailed morphological, molecular, physiological and genetic characterization of P23H-1 homozygous transgenic rats as an experimental model with visual and auditory dysfunctions is necessary.

## Author contributions

All the authors have made substantial contributions to the paper for having credit as authors. The individual tasks of the authors were as follows: conception of the work (Elena Caminos); design of the experiments, acquisition, analysis or interpretation of the data (all the authors: ABR experiments: Juan C. Alvarado; Immunocytochemical experiments in the cochlear nuclei: Jorge V. Sotoca and Elena Caminos; Auditory test: Jorge V. Sotoca and Elena Caminos; Statistical data analyses: Juan R. Martinez-Galan and Elena Caminos; Histology and immunocytology of cochleae: Verónica Fuentes-Santamaría. Drafting the work (Jorge V. Sotoca and Elena Caminos); revising it critically for important intellectual content (all the authors). Final approval of the version to be published (all the authors). Agreement to be accountable for all aspect of the work in ensuring that questions related to the accuracy or integrity of any part of the work have been appropriately investigated and resolved (Elena Caminos).

### Conflict of interest statement

The authors declare that the research was conducted in the absence of any commercial or financial relationships that could be construed as a potential conflict of interest.
